# Phytonutrients in Diseases of Affluence: Advances, Mechanisms, and Therapeutic Perspectives

**DOI:** 10.3390/nu18142297

**Published:** 2026-07-14

**Authors:** Katarzyna Zalewska, Barbara Zalewska, Przemysław Zalewski

**Affiliations:** Department of Pharmacognosy and Biomaterials, Poznan University of Medical Sciences, Rokietnicka 3, 60-806 Poznan, Poland

## 1. Introduction

Diseases of affluence, including obesity, type 2 diabetes mellitus, cardiovascular diseases, metabolic dysfunction-associated steatotic liver disease (MASLD), inflammatory bowel diseases, and selected cancers, represent some of the most significant public health challenges worldwide. The growing prevalence of these conditions has stimulated intense interest in dietary strategies and naturally occurring bioactive compounds that may contribute to prevention and treatment. Among these compounds, phytonutrients have attracted particular attention due to their pleiotropic biological activities, including antioxidant, anti-inflammatory, metabolic, and immunomodulatory effects.

This Special Issue, “Phytonutrients in Diseases of Affluence”, was established to provide a platform for disseminating current evidence regarding the role of plant-derived bioactive compounds in the prevention and management of chronic non-communicable diseases. The collection brings together original research articles, clinical observations, and comprehensive reviews addressing molecular mechanisms, preclinical evidence, and clinical applications of various phytonutrients. Collectively, the published papers highlight the diversity of bioactive compounds found in plant foods and their potential contribution to improving metabolic health and reducing the burden of modern lifestyle-related diseases.

## 2. Overview of Contributions

This Special Issue includes eight articles investigating different classes of phytonutrients and their implications for human health.

In the original research article, *“Nobiletin Protects Endothelial Function in High-Fat Diet-Induced Obese Mice Through Activation of 5′ Adenosine Monophosphate-Activated Protein Kinase”*, the authors demonstrated that nobiletin, a polymethoxylated flavonoid predominantly found in citrus fruits, exerts beneficial effects on vascular function in obesity [contribution 1]. The study identified activation of AMP-activated protein kinase (AMPK) as a central mechanism underlying improvements in endothelial function. Given the critical role of endothelial dysfunction in cardiovascular disease development, these findings provide valuable insight into potential nutritional approaches targeting obesity-related vascular complications.

Another experimental study, *“Sulforaphane Against the Metabolic Consequences of a High-Glycemic-Index Diet: Protective and Therapeutic Mechanisms Associated with Obesity and Insulin Resistance”*, explored the effects of sulforaphane, an isothiocyanate derived from cruciferous vegetables [contribution 2]. The findings highlighted the ability of sulforaphane to counteract adverse metabolic consequences associated with high-glycemic dietary patterns. The reported mechanisms involved improvements in insulin sensitivity, regulation of energy metabolism, and attenuation of obesity-related disturbances, supporting the growing interest in cruciferous vegetable-derived compounds as metabolic modulators.

Mental health aspects of phytonutrient intake were addressed in *“Polyphenol Consumption and Its Association with Physical and Mental Health in Adults with Major Depressive Disorder”*. This study expanded the traditional focus on metabolic diseases by examining the relationship between dietary polyphenol intake and both physical and psychological well-being in individuals with major depressive disorder [contribution 3]. The findings contribute to the emerging field linking nutrition, phytochemicals, and mental health outcomes, emphasizing the importance of dietary quality in comprehensive disease management.

Clinical evidence was represented by *“Clinical Outcomes of Oat Beta-Glucan Nutritional Intervention in Ulcerative Colitis: Case Reports of a Female and a Male Patient”*. These case reports examined the effects of oat-derived beta-glucans in patients with ulcerative colitis. Although based on a limited number of participants, the observations provide valuable preliminary insights into the potential role of dietary fiber and bioactive cereal components in supporting intestinal health and managing inflammatory bowel conditions [contribution 4].

Cancer prevention and treatment remain important areas of phytonutrient research. The review entitled *“The Chemopreventive and Anticancer Potential of Glucosinolates and Their Hydrolysis Products from Cruciferous Vegetables”* comprehensively summarized current knowledge regarding glucosinolates and biologically active metabolites such as isothiocyanates. The authors discussed mechanisms including modulation of carcinogen metabolism, induction of apoptosis, inhibition of tumor progression, and regulation of inflammatory pathways. The review reinforces the significance of cruciferous vegetables as important dietary components in cancer prevention strategies [contribution 5].

Liver diseases were the focus of two contributions. In *“Mechanistic Analysis of Fisetin in Liver Diseases and Its Potential Therapeutic Application in IFALD—A Review of In Vitro and In Vivo Studies”*, the authors evaluated the therapeutic potential of fisetin, a flavonoid present in various fruits and vegetables. The review highlighted anti-inflammatory, antioxidant, and hepatoprotective properties of fisetin and discussed its possible role in intestinal failure-associated liver disease (IFALD). The synthesis of available evidence suggests promising opportunities for future translational and clinical investigations [contribution 6].

Similarly, *“Chlorogenic Acid’s Role in Metabolic Health: Mechanisms and Therapeutic Potential”* reviewed evidence concerning one of the most abundant dietary polyphenols. Chlorogenic acid, commonly found in coffee and numerous plant foods, was shown to influence glucose metabolism, lipid homeostasis, oxidative stress, and inflammatory responses. The review provided a comprehensive overview of molecular pathways that may explain the observed associations between chlorogenic acid consumption and improved metabolic outcomes [contribution 7].

The final article, *“Influence of Matcha and Tea Catechins on the Progression of Metabolic Dysfunction-Associated Steatotic Liver Disease (MASLD)—A Review of Patient Trials and Animal Studies”*, examined the role of tea-derived catechins in liver health. By integrating evidence from both clinical and preclinical studies, the authors demonstrated the potential of matcha and tea catechins to influence hepatic lipid accumulation, oxidative stress, inflammation, and metabolic regulation. Given the increasing global prevalence of MASLD, these findings have important implications for future nutritional interventions [contribution 8].

Together, these contributions illustrate the broad spectrum of phytonutrients and disease targets represented within this Special Issue. Despite differences in study design and investigated compounds, several recurring themes emerge, including modulation of oxidative stress, attenuation of chronic inflammation, improvement of metabolic regulation, and protection of vascular and hepatic function.

## 3. Future Perspectives

The studies included in this Special Issue underscore the growing importance of phytonutrient research in addressing diseases of affluence. While substantial progress has been made in elucidating molecular mechanisms and demonstrating beneficial effects in experimental models, several challenges remain. Future investigations should focus on well-designed randomized clinical trials, standardized phytonutrient preparations, dose–response relationships, bioavailability, and long-term safety. Advances in metabolomics, nutrigenomics, and precision nutrition may further facilitate the identification of population groups most likely to benefit from specific phytonutrient interventions.

An additional priority involves translating promising laboratory findings into practical dietary recommendations and evidence-based clinical applications. Greater integration of mechanistic, clinical, and epidemiological research will be necessary to establish robust nutritional strategies capable of reducing the burden of chronic diseases worldwide.

## 4. Conclusions

This Special Issue, “Phytonutrients in Diseases of Affluence”, provides a comprehensive overview of contemporary research examining the health-promoting properties of plant-derived bioactive compounds. The collected studies demonstrate the considerable potential of phytonutrients, including nobiletin, sulforaphane, polyphenols, beta-glucans, glucosinolates, fisetin, chlorogenic acid, and tea catechins, to influence key biological pathways involved in metabolic, cardiovascular, inflammatory, hepatic, and neoplastic diseases. Together, these contributions strengthen the scientific foundation supporting the role of phytonutrient-rich dietary patterns in disease prevention and management and offer valuable directions for future research.

